# Newborn with Dilated Cardiomyopathy Secondary to Vitamin D Deficiency

**DOI:** 10.1155/2012/945437

**Published:** 2012-09-26

**Authors:** Hanan Al Azkawi, Angham Al Mutair

**Affiliations:** Pediatric Endocrinology Division, King Abdulaziz Medical City, Riyadh, Saudi Arabia

## Abstract

Hypocalcemia is a rare but reversible cause of dilated cardiomyopathy with limited cases being reported in the literature. Vitamin D deficiency is the main cause of hypocalcemia in almost all reported cases. We report a newborn presented with hypocalcemia-induced dilated cardiomyopathy secondary to vitamin D deficiency. After calcium and vitamin D therapy, the baby showed a rapid recovery of the cardiac function.

## 1. Introduction

Dilated cardiomyopathy (DCM) has an estimated incidence of 1.13 cases per 100,000 children. Diagnosing the primary etiology occurs in fewer than half of these children but significantly improves their outcome [1]. DCM mostly idiopathic, however, infection and metabolic causes has been identified in some cases in which the defect in myocardial contractility is irreversible [2].

Here, we report a case of dilated cardiomyopathy secondary to hypocalcemia that originated from a nutritional vitamin D deficiency induced rickets in newborn who was exclusively breastfed by a mother who had vitamin D deficiency.

## 2. Case

RA is now, 1-year-old boy who was full term, spontaneous vaginal delivery with birth weight of 3.00 kilogram (kg); at 25th percentile, length 54 centimeter (cm); at 90th percentile and head circumference 34 cm; at 25th percentile and he was discharged home in the second day of life with no medical problem. He presented at age of 35 days with poor/interrupted feeding, shortness of breath, tachycardia, and vomiting for 3 days. On examination, he has no dysmorphic features, he was tachycardiac; pulse rate 170/minute, tachypnoei; respiratory rate (RR) = 80/minute with blood pressure was 84/58, and was having cold extremities (capillary refilling time was more than 3 seconds). The oxygen saturation was less than 90% on room air, then improved to 100% on oxygen. At presentation his weight was 4.4 kg at 50th percentile, length was 57 cm at 90th percentile, and head circumference was 35.5 cm at 10th percentile. Cardiovascular examination revealed first and second heart sound were normal, loud third heart sound with a gallop rhythm (heard all over pericardium) more in the left lower sternal border. Apex is at six intercostals space, mid-clavicular line with positive right ventricle heaves. All pulses were felt with no radio femoral delay. On the chest exam there were bilateral basal crackles. Abdominal examination revealed hepatomegaly with liver span nine centimeter. Central nervous system (CNS) examination revealed sick baby, moving all limbs with no focal neurological deficit.

**Figure 1:**
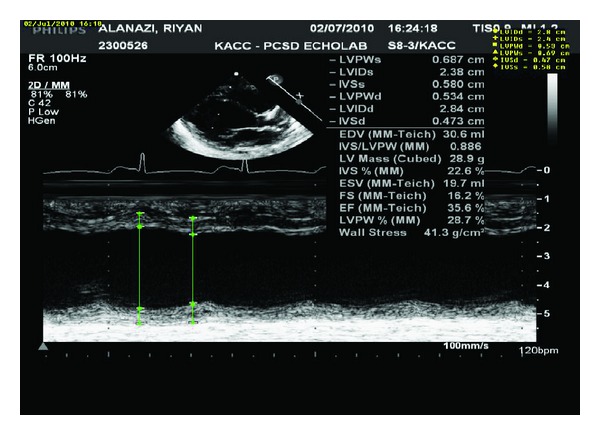
Echo for the patient showed depressed myocardiac function with ejection fraction 35.6%.

Initial impression was sepsis for which septic work up done including blood, urine and cerebrospinal fluid cultures which then came to be negative. The baby was resuscitated and managed by intravenous fluid, oxygen, as well as started on antibiotic {cefotaxim (30 mg/kg/dose) every eight hours intravenously} which was stopped based on the negative cultures result. His initial investigations; chest X-ray revealed significant cardiomegaly (80% of cardiothoracic ratio) and congested pulmonary vessel, electrocardiography (ECG) showed prolonged QT interval, and echocardiogram (Echo) revealed dilated left ventricle with moderate to severe depressed left ventricular function and ejection fraction was 35.6%, has moderate mitral valve regurgitation and no pericardial effusion (Figure 1).

The impression by the cardiologist was dilated cardiomyopathy (DCM) with possibility of myocarditis. Antifailure medications was started these include frusemide started with 3 mg then increased to 4 mg after 3 days every eight hours intravenously (1 mg/kg/dose), captopril started with 0.5 mg every eight hours orally and gradually build up and reach maximum dose of 2 mg every eight hours on day nine after admission orally, and digoxin 0.02 mg twice per day orally was stared on day eleven after admission. One dose of immunoglobulin empirically was given at dose of 4 gram intravenous infusion. Serology was sent for coxsackie virus antibodies (B1–B6, A9) and EBV profile which all came to be negative. 

RA also fully evaluated by metabolic team and was started empirically on carnitine 200 mg twice per day (50 mg/kg/dose). His metabolic work up was normal, including; ammonia 73 unitmol per litter (normal 18–72 unitmol/littr), lactic acid 2.8 mmol per litter (0.5–2.2 mmol/litter), repeated after 3 days and was normal 1.0 mmol/litter, neonatal screen by tandem mass spectrometry and gas chromatography mass spectrometry for urine organic aciduria were negative, L-carnitine level was within normal; 33.4 micromol per litter (normal 38.1–68.0 micromol/litter) done twice, pyruvate was normal; 47.1 micromol per litter (normal 60.0–100 micromol/litter), and carbohydrate-deficient transferring for congenital glycosylation was also negative 0.60% (normal <1.75%). Other blood investigations were within normal including, complete blood count, liver, and renal profiles (Table 1).

Six days after admission the baby did not improve rather he became more sick required mechanical ventilation. Blood investigations which were sent showed severe hypocalcemia, total calcium 1.13 mmol/L (normal 2.25–2.75 mmol/L), high phosphorus Po4 2.69 mmol/L (normal 0.74–1.52 mmol/L); which can be explained by poor tissue perfusion due to poor cardiac output, high alkaline phosphatase 1315 U/L (normal <500 U/L), normal magnesium Mg 0.75 mmol/L (normal 0.71–0.95 mmol/L), normal Albumin 39 g/L (normal 38–54 G/L) and high parathyroid hormone 32 pmol/L (normal 1.60–7.20 Pmol/L) (AEROSET and ARCHITECT c8000 systems) (Table 2). Vitamin D3 level came very low less than 4 nmol/L (<37 nmol/L: severe deficiency, 37–75 nmol/L mild to moderate deficiency or insufficient and 76–200 nmol/L optimum level) (immunoassay technique 38038 using HPLC system and LC-MS/MS). 

Mother of RA is 25 years old, multigravidae with two other children one is three years old and another one is 18 months old. No similar history. Evaluation of mother's vitamin D level revealed severely deficient, level was less than 3 nmol/L (<37 nmol/L: severe deficiency, 37–75 nmol/L mild to moderate deficiency or insufficient and 76–200 nmol/L optimum level). During her pregnancy she was not on vitamin D supplement. Her adjusted calcium was low normal 2.25 mmol/L (normal 2.10–2.55 mmol/L) and alkaline phosphatase was high 280 U/l (normal 40–150 U/L). 

**Figure 2:**
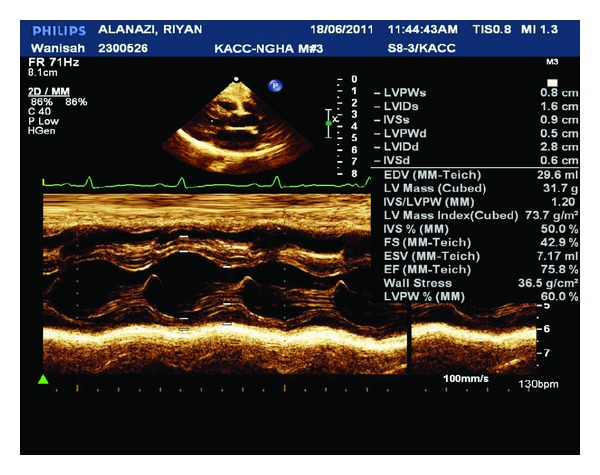
Echo for the patient showed improved myocardial function with ejection fraction 75.8%.

RA managed with intravenous calcium gluconate of 10% concentration initially as 2 mL per kg bolus over 30 minutes then as infusion (100 mg of elemental calcium diluted in 50 mL 5% dextrose fluid and given at rate of 2 mg/kg/hour). At same time he was started on oral calcium 80 mg/kg/day of elemental calcium in three divided doses and vitamin D3 (cholecalciferol) 3000 unit daily orally. He improved clinically as his serum calcium gradually improved and normalizes 2.41 mmol/L on day three of calcium infusion which was continuously tapered then stopped. The oral calcium dose also reduced based on the normal serum calcium level then stopped before discharge, at age of 50 days, and continued on vitamin D. The alkaline phosphatase also improved to 806 U/L. He was able to be weaned off ventilation after three days. The cardiac function also improved and the antifailure medications were tapered and then stopped; digoxin was stopped after three weeks, frusemide and captopril after two months and carnitine also stopped after one year when the L-carnitine level was repeated for the third time and came to be normal 48.2 micromol/L (normal 38.1–68.0 micromol/L). Repeated Echo showed improvement of the left ventricular contractility with EF of 50% then to 62% (day five, day ten after admission, resp.). Last echo after one year showed normal cardiac function with EF of 75% (Figure 2).

RA is now one year old, completely asymptomatic on maintenance dose of vitamin D3, 400 units daily orally and has normal growth and development.

## 3. Discussion 

Our case presented with sever hypocalcemia-induced dilated cardiomyopathy secondary to nutritional vitamin D deficiency at a very young age with rapid recovery of the cardiac function after calcium and vitamin D therapy.

Calcium has a central role in myocardial contraction coupling, hypocalcemia reduces myocardial function. Congestive cardiac failure due to hypocalcemia was reported, though rare and few cases of hypocalcemia-induced cardiomyopathy have been reported [3, 4]. Hypocalcemia causing DCM is reversible with complete recovery after normalization of serum calcium. Vitamin D deficiency is the main cause of hypocalcemia in infants and old children. Nutritional rickets is still prevalent with the primary etiology being vitamin D deficiency in the breastfed infants and children [1, 5].

Ionized calcium has a central role for regulating myocardial contraction. During the cardiac action potential is activated, ionized calcium enter intracellular through depolarization-activated calcium channels. Entered ionized calcium triggers calcium release from the sarcoplasmic reticulum (SR). Ca^2+^ bind to the myofilaments proteins such as troponin C initiate contraction of myocardium [6, 7]. There are two main ways to change contractility of myocardium. One is altering of amplitude or duration of Ca^2+^ transient; another is altering of sensitivity of the myofilaments to Ca^2+^ [6, 7]. Therefore, hypocalcemia-induced DCMP is developed by the altering of amplitude or duration of Ca^2+^ transient [8, 9]. 

Vitamin D deficiency remains a major public health problem in the Middle East especially among infants who are exclusively breastfed and born to mothers with high-risk factors such as low vitamin D stores, dark skinned, and/or living a sedentary lifestyle which further limit adequate ultraviolet light exposure [10–12]. Infants born to mothers with deficiency of vitamin D are at risk of developing early and fatal squeals of hypocalcemic vitamin D deficiency [13–15].

In our case, the baby presented with severe vitamin D deficiency resulting in severe hypocalcemic DCM at very young age, 4 weeks. He was exclusively breastfed. Evaluation of vitamin D status of his mother showed severe vitamin D deficiency. 

Recently, both the American Academy of Pediatrics (AAP) and the Canadian Pediatric Society (CPS) have revised the previous recommendation of a minimum daily intake of 200 IU/day of vitamin D beginning in the first two months after birth to a minimum daily intake of 400 IU beginning soon after birth, especially for infants whose mothers are vitamin D deficient or in those infants not exposed to adequate sunlight [16, 17]. Supplementation should be continued unless the infant is weaned to at least one liter/day of vitamin D-fortified formula. In addition, relatively high-dose maternal vitamin D supplements (2000 IU/day), together with maintaining a healthy, balanced diet is needed [16, 17]. 

This new guideline should be emphasized as part of the educational program to all pregnant and lactating mothers, especially those who are at risk for vitamin D deficiency [18].

In one study a hospital database search was conducted from year 1997 to year 2007 to identify patients with confirmed vitamin D deficiency in addition to DCM. Four exclusively breastfed African American infants were identified. These infants presented with congestive heart failure secondary to DCM and, at their admission, were found to have laboratory evidence consistent with hypocalcemic rickets. These patients responded dramatically to treatment with vitamin D and calcium, and cardiac function returned to normal within months [13].

In another series, 15 Indian infants (age between 45 days to 5 months) presented with severe left ventricular dysfunction, who were found to have hypocalcemia with or without hypomagnesemia. Vitamin D deficiency was identified as the main cause of hypocalcemia. These children improved on supplementation of vitamin D and calcium [19].

This baby was investigated for other possible causes for his DCM. There was negative family history of similar problem. Investigations were negative for metabolic disorders known to cause DCM (e.g., carnitine deficiency). Others like infection-related DCM also was looked for in this baby and was negative for most known viruses (coxsackie virus and EBV). Echo showed no other cardiac defect other than moderate to severe dilated cardiomyopathy.

Our case demonstrates severe hypocalcemic-induced dilated cardiomyopathy secondary to nutritional vitamin D deficiency.

RA when treated with calcium and vitamin D he improved dramatically and was able to wean ventilation and stop the antifailure medications. His cardiac function returned to normal. He is now one year old, completely asymptomatic on maintenance dose of vitamin D3. Six months after followup the parathyroid hormone normalize 3.16 pmol/L (normal 1.60–7.20 pmol/L). Alkaline phosphatase also return to normal after two months of treatment 301 U/L (normal <500 U/L). Vitamin D level also improved to 64 nmol/L then normalizes 77 nmol/L (at two months and six months resp.). To best of our knowledge this is the first case reported in the Gulf region and Saudi Arabia for hypocalcemic-induced dilated cardiomyopathy with complete recovery.

## 4. Conclusion 

This association of hypocalcemia-induced DCM is of noted importance when encountering a patient with a new diagnosis of DCM. With increased suspicion, the diagnosis of hypocalcemia-induced DCM can be made and prompt treatment can lead to recovery cardiac function and better patient outcome. This report adds to the already existing evidence that vitamin D deficiency remains a major health problem in Saudi infants, especially for mothers who already have low vitamin D stores due to cultural, nutritional, or health style risk factors. This paper highlight the importance of implanting the compliance with vitamin D prophylaxis for pregnant mothers at daily dose of 800 unit–1000 unit and for the babies at daily dose of 400 unit soon after birth. This can prevent hypocalcemia induced dilated cardiomyopathy which is a serious complication of nutritional vitamin D deficiency.

## Figures and Tables

**Table 1:** Complete blood count, liver, and renal profile and for the patient at presentation, 6 and 9 days after admission and at discharge.

		At presentation	6 days after admission (when got sick)	Day 9 of admission	At discharge
Liver profile	T. bil (<205 UMOL/L)	20.0	37	12.9	
	Alb (38–54 G/L)	37	34	38	37
	AST (5–34 U/L)	27	44	25	29
	ALT (5–55 U/L)	14	18	14	20
	ALK (<500 U/L)	1483	1315	806	648
	GGT (12–64 U/L)	20	19	16	29

Renal profile	Na (138–145 MMOL/L)	139	135	137	136
	K (4.1–5.3 MMOL/L)	4.0	5.0	4.1	4.0
	CL (95–110 MMOL/L)	103	101	102	108
	CO_2_(20-28 MMOL/L)	16	20	21	20
	Urea (1.1–8.0 MMOL/L)	2.2	2.7	1.1	3.2
	Creatinine (18–35 UMOL/L)	38	41	37	32
	Glucose (2.8–4.4 MMOL/L)	5.5	5.9	5.2	4.8

CBC	HB (106–145 G/L)	118	77	138	136
	WBC (6.0–16.0)10^*∧*^G/L	16.2	7.6	10.6	12.8
	Plt (150–450)10^*∧*^G/L	498	462	411	483

**Table 2:** Bone profile and parathyroid hormone for the patient on day 6 and 9 after admission and at discharge.

	At presentation	6 days after admission (when got sick)	Day 9 of admission	At discharge
PTH (1.60–7.20 PMOL/L)		32.50		
T. Ca (2.25–2.75 MMO/L)		1.13	2.41	2.46
Adjusted Ca (2.25–2.75 MMOL/L)		1.25	2.45	2.65
Mg (0.71–0.95 MMOL/L)		0.75	0.97	1.09
PO_4_(0.74–1.52 MMOL/L)		2.69	1.60	1.06

## Abbreviations

T. bil: Total bilirubin
Alb: Albumin
AST: Aspartate transaminase
ALT: Alanin transaminase
ALK: Alkalin phosphatase
GGT: Gamma glutamyl transferase
Na: Sodium
K: Potassium
CL: Chloride
CO_2_: Carbon dioxide
HB: Hemoglobin
WBC: White blood cell
Plt: Platelet
PTH: Parathyroid hormone
T. Ca: Total calcium
Mg: Magnesium
PO_4_: Posphorus.
